# A survey of the causes of cattle organs and/or carcass condemnation, financial losses and magnitude of foetal wastage at an abattoir in Dodoma, Tanzania

**DOI:** 10.4102/ojvr.v82i1.855

**Published:** 2015-04-16

**Authors:** Wilfred Tembo, Hezron E. Nonga

**Affiliations:** 1 Department of Veterinary Medicine and Public Health, Sokoine University of Agriculture, Tanzania; 2 Department of Veterinary Services, Ministry of Agriculture and Livestock, Zambia

## Abstract

Slaughterhouses provide a safeguard that prevents the public from consuming meat of poor quality or meat which may be infected with zoonotic diseases. This work reviews a 3-year database of cattle that were slaughtered and inspected between 2010 and 2012 at Dodoma abattoir, Tanzania. In addition, meat inspection was undertaken for 1 month (December 2013). The aim of this study was to establish causes of organ and carcass condemnations and their financial implications as well as the magnitude of slaughter of pregnant cows at Dodoma abattoir. During retrospective study, it was found that a total of 9015 (10.5%) lungs, 6276 (7.3%) intestines, 5402 (6.3%) livers, 3291 (3.8%) kidneys and 41 (0.05%) carcasses were condemned. Pulmonary emphysema (3.4%), fasciolosis (4.5%), pimply gut (5.7%), kidney congenital cysts (1.9%) and hydatidosis (3.1%) were major causes of organ condemnations. This large number of condemned edible organs and/or carcasses implies that public health considerations result in deprivation of valuable protein. Occurrence of hydatidosis, cysticercosis, fasciolosis and tuberculosis illustrates the possible public health problem and presence of environmental infections. Of the 794 cows slaughtered in December 2013, 46% were pregnant. Financial loss as a result of organ and/or carcass condemnations was estimated at $9892. Condemnation of organs and/or carcasses and indiscriminate slaughter of pregnant cows represent a significant loss of meat and revenue and a reduction in growth of future herds, which has a negative effect on the livestock industry. This justifies appropriate surveillance and disease control programmes coupled with strict enforcement of legislation governing animal welfare to curb the slaughter of pregnant animals.

## Introduction

Tanzania Mainland has about 50 million ha of land suitable for grazing and has the third largest livestock population in Africa, after Sudan and Ethiopia (Ministry of Livestock Development and Fisheries [MLDF] 2012). Tanzania has a total of 21 400 889 cattle, 99.4% of which are reared by smallholder farmers (MLDF 2012). This places beef production amongst the most important economic and rural livelihood activities in Tanzania. Indeed, indigenous cattle provide more than 70% of meat as well as 67% of milk consumed in Tanzania (Mellau, Nonga & Karimuribo 2010a). The contribution of livestock is not limited to its share in the total gross domestic product (GDP); it also contributes to the national food supply (meat, eggs, milk) and to crop production through the provision of manure and draught power (MLDF 2012). However, the potential contribution of livestock cannot be fully exploited because of losses that occur as a result of morbidity and mortality as a result of livestock diseases. Some of these losses can only be observed at slaughterhouses during antemortem and postmortem examinations. The number of organs and carcasses condemned for various reasons implies serious economic losses to farmers and the livestock industry in the country (Mellau, Nonga & Karimuribo 2011). In addition, condemnations also reduce the availability of meat required by the human population to meet their protein and mineral requirements.

Apart from the losses resulting from condemnations, others occur as a result of slaughter of pregnant cows, a situation that obviously adds to the bulk of losses to the farmer and the livestock industry as a whole. In view of the increasing human population, Tanzania requires a corresponding increase in its cattle population and improved herd health management. However, the increase in cattle population cannot be fully attained because of foetal losses that occur as a result of slaughter of pregnant cows. Previous studies have found that up to 8.6% of cows slaughtered in Arusha were pregnant (Mellau, Nonga & Karimuribo 2011) whilst 14.4% cows slaughtered at Yola modern abattoir in Adamawa State, Nigeria were pregnant (Ardo, Lawal & Aliyara 2013). Slaughter of pregnant cows at slaughterhouses threatens the growth of the cattle population with serious repercussions for any country's livestock industry.

Dodoma is one of the regions with a high number of livestock in Tanzania. It is a capital city where all parliamentary sessions and many important government and political party activities are undertaken. Dodoma abattoir is the main source of beef for all the delegates to these functions, as well as for the general population of the municipality. Dodoma region is amongst the drier areas of Tanzania with variable climatic conditions and unpredictable rainfall, which may have a direct influence on the patterns of livestock diseases. However, there has been no study on diseases of cattle raised in such drier areas of Tanzania. The quality and safety of beef consumed in Dodoma is unknown, as there has been no study on common pathological lesions encountered in slaughtered cattle. Causes of organ and carcass condemnations and the associated financial implications have not been documented. Although similar surveys have been conducted in other regions, it is important to carry out the study because the prevalence and incidence of diseases differ according to geographical areas. Furthermore, such data could be a convenient and inexpensive source of information that could be used to monitor disease trends and possible emergence and re-emergence of pathogens. Moreover, the extent of slaughter of pregnant cows in the region is not known although there have been limited reports in some areas of Tanzania (Maro 2014; Mellau, Nonga & Karimuribo 2011). The aim of this study was to provide baseline data on the common causes of slaughterhouse condemnations of organs and/or carcasses and to estimate the financial losses that occur at Dodoma abattoir.

## Materials and methods

### Study area and animals

The survey was conducted at an abattoir in Dodoma municipality, which lies between latitude 6.1731°S and longitude 35.749°E. The municipality has a human population of 410 956 (Population and Housing Census of Tanzania [PHCT] 2013). Dodoma region covers an area of 2669 km^2^ and has 2 083 588 people (PHCT 2013) and about 1 185 501 cattle (MLDF 2012). It receives a mean annual rainfall of less than 570 mm and the wet season starts in November and ends in May. The mean minimum and maximum temperatures vary according to altitude but average at 16 ° C and 29 ° C, respectively. The bulk of the cattle slaughtered come from Dodoma itself and the adjoining areas of Arusha and Singida regions. Dodoma abattoir is owned by Tanzania Meat Company and it slaughters an average of 81 cattle per day.

### Study design and data collection

This study involved the use of both retrospective (secondary) and prospective (primary) data. The retrospective study involved retrieval of slaughter records of a 3-year period from 2010 to 2012. As a means of quality control of data, all records with no proper diagnosis of organ and/or carcass lesions and ambiguous information on species and slaughter dates were excluded from the study. Routine meat inspection is carried out by qualified meat inspectors with a diploma in Animal Health and Production who have undertaken special training in meat inspection, meat processing and pathology of farm animals. The meat inspectors perform their work under the supervision of qualified veterinarians. During routine meat inspection, the disease conditions were grossly diagnosed based on pathological changes such as colour, size, morphology, consistency and presence of lesions or parasites as described by Gracey (1986). Judgement on whether organs and/or carcasses were fit or unfit for human consumption was based on guidelines provided by Tanzania's Meat Inspection Guidelines (United Republic of Tanzania [URT] 1962) and as described by Gracey (1986) and the Food and Agriculture Organization of the United Nations (FAO) (1994). At the end of meat inspection every day, all partially and totally condemned carcasses and organs were taken to the abattoir laboratory for further examination and identification of the lesions and parasites, if any. In case of doubt, if lesions would need further investigation and as a means of external validation, the abattoir submitted samples to Dodoma Veterinary Investigation Centre (VIC) for diagnostic confirmation.

Collection of prospective primary data was carried out through active meat inspection conducted in December 2013 using the same approach as for the retrospective study. Estimation of foetal loss was arrived at by observation and palpation of uteri of all slaughtered cows.

### Estimation of financial loss attributed to organ and/or carcass condemnations

Estimation of financial loss attributed to organ and/or carcass condemnations was established based on the data recorded in December 2013. The parameters used for assessing the financial loss were the average weight of organs and/or carcasses in kilograms and average prices per kilogram of organs and/or carcasses in US dollars ($). Financial loss for the 1-month period was established by direct computation of the product (by multiplication) of the average weight of one organ or carcass, the average price (per kg of organ or carcass) and the total number of condemned organs and/or carcasses, as described by Mwabonimana *et al*. (2009). The annual financial loss was obtained by assuming that the parameters (organ and carcass condemnation rates per month and average meat prices) remained relatively constant during the year.

### Data analysis

The data were entered into a Microsoft Excel spread-sheet and the proportions (%) of lesions were calculated considering the number affected against the total number of animals slaughtered and inspected. A chi squared test was used to compare the proportions (%) of lesions obtained in the wet and dry seasons and at a critical probability of *p* < 0.05 using the Stat Calc function on Epi Info™ (Version 7, Centers for Disease Control, Atlanta, USA). Furthermore, foetal losses were quantified as proportion (%) of the cows’ uteri that were found with foetuses over the total number of cows that were slaughtered during the prospective study.

## Results

### Slaughtered cattle and conditions in retrospective study

Based on meat inspection records, a total of 85 980 cattle were slaughtered and inspected at Dodoma abattoir during the period 2010–2012. Of the slaughtered cattle, 29.4% (*n* = 25 298) had pathological conditions that lead to condemnations of organs and/or carcasses (Table 1). A total of 667 (0.8%) carcasses had lesions or pathological conditions and of these, 41 (0.05%) were totally condemned because of jaundice (0.03%; *n* = 27), cysticercosis (0.01%; *n* = 12) and tuberculosis (0.002%; *n* = 2). The rest were provisionally passed for human consumption after being chilled for 10 days at -10 ° C for cases of cysticercosis, chilled for 24 h for cases of mild jaundice and mild haemorrhages, and trimmed for bruises.

**TABLE 1:** Number and percentage of organs and/or carcasses condemned according to condition at Dodoma abattoir, Tanzania for the period 2010–2012.

Organs/carcasses	Conditions	2010 (*n* = 27 444)		2011 (*n* = 30 671)		2012 (*n* = 27 865)		Total (*n* = 85 980)	
		Number	%	Number	%	Number	%	Number	%
Carcasses condemned	Jaundice, cysticercosis and tuberculosis	10	0.04	16	0.05	15	0.05	41	0.05
Livers	Fasciolosis	1327	4.8	1293	4.2	1283	4.6	3903	4.5
	Calcified cysts	292	1.1	241	0.8	347	1.3	880	1.0
	Peritonitis	60	0.2	47	0.2	49	0.2	156	0.2
	Abscesses	46	0.2	46	0.2	17	0.1	109	0.1
	Hydatidosis	17	0.1	39	0.1	115	0.4	171	0.2
	Others (hepatomegaly, telengiactasis, hepatitis)	53	0.2	64	0.2	66	0.2	183	0.2
Lungs	Emphysema	788	2.9	853	2.8	1315	4.7	2956	3.4
	Hydatidosis	766	2.8	677	2.2	1016	3.7	2459	2.9
	Haemorrhages	542	2.0	476	1.6	953	3.4	1971	2.3
	Pleurisy	183	0.7	165	0.5	247	0.9	595	0.7
	Pneumonia	90	0.3	198	0.7	480	1.7	768	0.9
	Calcified cysts	49	0.2	79	0.3	120	0.4	248	0.3
	Abscesses	9	0.03	5	0.02	0	0.0	14	0.02
	Tuberculosis	1	0.004	0	0.0	2	0.01	3	0.003
	Congestion	1	0.004	0	0.0	0	0.0	1	0.001
Aortas	Onchocercosis	17	0.1	119	0.4	274	1.0	410	0.5
Kidneys	Congenital cysts	273	1.0	457	1.5	871	3.1	1601	1.9
	Hydronephrosis	221	0.8	219	0.7	588	2.1	1028	1.2
	Infarcts	78	0.3	42	0.1	50	0.2	170	0.2
	Renal calculi	78	0.3	66	0.2	77	0.3	221	0.3
	Others (nephritis, necrosis, melanosis)	91	0.3	138	0.5	42	0.2	271	0.3
Hearts	Calcified cysts	111	0.4	102	0.3	116	0.4	329	0.4
	Pericarditis	53	0.2	49	0.2	123	0.4	225	0.3
	Cysticercosis	20	0.1	0	0.0	6	0.02	26	0.03
Plucks	Tuberculosis	4	0.01	0	0.0	0	0.0	4	0.01
	Abscesses	7	0.03	11	0.04	18	0.1	36	0.04
	Pleurisy	12	0.04	22	0.1	9	0.03	43	0.05
Intestines	Pimply gut	1445	5.3	1733	5.7	1720	6.2	4898	5.7
	Enteritis	340	1.2	311	1.01	644	2.3	1295	1.5
	Peritonitis	15	0.1	44	0.1	13	0.1	72	0.1
	Abscesses	7	0.03	2	0.01	2	0.01	11	0.01
Spleens	Splenomegaly	91	0.3	40	0.1	52	0.2	183	0.2
	Peritonitis	6	0.02	2	0.01	0	0.0	8	0.01
	Abscesses	3	0.01	1	0.003	4	0.01	8	0.01
Heads	Abscesses	1	0.003	0	0.0	0	0.0	1	0.001
**Total**	**-**	**7291**	**26.6**	**7796**	**25.4**	**10837**	**38.9**	**25924**	**30.2**

Of all the cattle that were slaughtered during the 3-year period, a total of 25 924 (30.2%) organs and/or carcasses were condemned. Organ-specific condemnations were: 9015 lungs (10.5%) – the highest number of condemnations – followed by 6276 (24.8%) intestines, 5402 (21.4%) livers and 3291 (13%) kidneys, 580 (0.7%) hearts and 410 (0.5%) aortas (Table 1). The least frequently condemned organs were the spleen (0.8%), plucks (0.3%) and head (0.004%). Of the lungs that were condemned, emphysema (32.8%) was the leading cause of condemnations, followed by hydatidosis (27.3%) and haemorrhages (21.9%). Lumps in the gut as a result of oesophagostomosis, i.e. pimply gut (78%), was the main cause of condemnations of intestines. Fasciolosis accounted for 72.3% of condemned liver followed by calcified cysts (16.3%). Of 3291 kidneys that were condemned, congenital cysts (48.7%) and hydronephrosis (31.2%) were the main reasons for condemnation.

It was further found that a total of 49 571 (57.7%) and 36 409 (42.4%) cattle were slaughtered in the wet and dry seasons, respectively. Significant differences (*p* < 0.05) were observed in the occurrence of some conditions between the wet and dry seasons. Calcified cysts, peritonitis, abscesses, pimply gut and splenomegaly were more prevalent during the wet seasons. On the other hand, jaundice, bruises, emphysema, lung haemorrhages, hydronephrosis, renal calculi, onchocercosis and pericarditis were more prominent in the dry seasons. No significant differences (*p* > 0.05) were observed in other conditions between the wet and dry seasons.

### Slaughtered cattle and conditions in December 2013

In the primary data, 2438 cattle were slaughtered and inspected, of which 794 (32.6%) were cows, 641 (26.3%) were bulls and 1003 (41.1%) were castrates. A total of 2329 (95.5%) cattle were the common local shorthorn Zebu cattle whilst the rest (4.5%) were crossbreeds of exotic dairy and beef cattle. The stock movement permits at the abattoir indicated that most of the cattle (98%) came from Kizota livestock market in Dodoma whilst the rest (2%) came from individual suppliers who delivered cattle directly to the abattoir. Even though 47 (1.9%) of cattle were emaciated and 446 (18.3%) were soiled with dung and mud, no animal was condemned at antemortem inspection during the prospective study.

Of all the cattle that were slaughtered in December 2013, 1247 (51.1%) had diseases or conditions that led to condemnations. One carcass was totally condemned because of generalised tuberculosis whilst another carcass was condemned as a result of cysticercosis. Organ assessment showed that 325 (13.3%) lungs, 186 (7.6%) livers, 279 (11.4%) intestines, 84 (3.4%) kidneys, 32 (1.3%) hearts and 334 (13.7%) aortas were condemned. Onchocercosis (13.7%) was responsible for all aortas that were condemned. The rest were pimply gut (10.2%), fasciolosis (4.1%), pulmonary emphysema (5.2%), calcified cysts (3.7%), congenital cysts (1.7%), hydatidosis (2.7%), enteritis (1.2%), pericarditis (0.5%) and cysticercosis (0.1%).

### Financial losses as a result of condemnations

The prices that were used in the estimation of financial losses were those that prevailed in Dodoma municipality during the prospective study. The average weight of organs and/or carcasses, total number of organs and/or carcasses condemned, average unit cost per kg of organs and/or carcasses and the total financial losses attributable to organ and carcass condemnations are indicated in Table 2. The total cost of all organs and carcasses during 1 month was $9892. Assuming a relatively constant rate of condemnations and prices of meat, the annual financial loss was estimated at $118 702.

**TABLE 2:** Financial loss in prospective study carried out in December 2013 at Dodoma abattoir, Tanzania.

Organs/carcasses	Number of organs/carcasses condemned	Average weight of organs/carcasses (kg)	Average unit cost in $	Total loss in $	Total annual loss in $
Carcasses	2	180	4.6	1636.40	19 636.40
Livers	186	3.2	4.6	2705.50	32 465.50
Lungs	325	2.8	2.0	1772.70	21 272.70
Aortas	334	0.3	2.0	195.20	2342.40
Kidneys	84	0.2	4.6	76.40	916.40
Hearts	32	0.6	4.6	87.30	1047.30
Intestines	279	6.2	2.0	3369.80	40 436.90
Heads	5	1†	9.7	48.70	584.50
**Total**	**-**	**-**	**-**	**9891.90**	**118 701.80**

†, Price of heads was uniform regardless of the weight.

### Numbers of pregnant cows slaughtered

A total of 794 cows were slaughtered and their uteri examined for pregnancy. Of all cows slaughtered, 365 (46.0%) were found to be pregnant.

## Ethics statement

Permission to carry out this study was granted by the Dodoma Municipal Director and ethics approval for the study was given by the Ethical Committee of Sokoine University of Agriculture (SUA), Morogoro, Tanzania. The Vice Chancellor of SUA issued a research permit letter on behalf of the Tanzanian Commission for Science and Technology (COSTECH) that permitted the researcher from the University to conduct research at Dodoma municipal abattoir. The management of the abattoir also issued a permission letter for the abattoir records to be used for research and to conduct a 31-day survey of causes of organ and/or carcass condemnations, financial losses associated with those condemnations and the magnitude of slaughter of pregnant animals at the abattoir. Verbal consent was obtained from each of the cattle owners who had sent animals for slaughter at the abattoir after explaining the purpose and importance of the study prior to data collection.

## Discussion

The present study has revealed that a number of conditions result in the condemnation of organs and carcasses and thus have financial implications. Furthermore, the apparently prevalent slaughter of pregnant cows is of animal welfare concern. The condemnation of organs and carcasses reduces the availability of necessary protein, vitamins and minerals and deprives the farmers and cattle traders of valuable income. The existence of tuberculosis, hydatidosis, fasciolosis and cysticercosis, despite the low prevalence of some of these conditions, is an indication of a clear public health threat to meat handlers, livestock keepers and meat consumers. Therefore, the overall economic loss, the public health risk and the negative impact on the national livestock industry cannot be overemphasised.

Because of their anatomical and histological characteristics, lungs are perhaps the organs most exposed to physical, chemical and biological injuries. This is supported by the findings of the current study, which revealed that 35.6% of all condemned organs were lungs. This was higher than the results obtained in Arusha by Mellau, Nonga and Karimuribo (2011). Elsewhere, in Ismailia, Egypt, Ahmed, Ismail and Dessouki (2013) reported that lungs contributed up to 44.6% of all condemned organs. This is in agreement with findings by Mellau, Nonga and Karimuribo (2010b), who reported that emphysema had contributed 13.1% to organ condemnations at Arusha abattoir in Tanzania. Ruminants, particularly cattle, have well-developed interlobular septa and lack of collateral ventilation, making them more susceptible to interstitial emphysema (Mellau, Nonga & Karimuribo 2010b). Pulmonary emphysema is associated with diseases such as East Coast fever; it may also be caused by obstruction of airflow or by extensive gasping respiration during the slaughter process (FAO 1994). Improper stunning, delayed slaughter after stunning and delayed hoisting after slaughter may also have contributed to the high number of lungs with emphysema and haemorrhages (personal observation). Exposure of animals to stress factors like dust, overcrowding and exhaustion from long treks in search of pasture and water during the dry season may also contribute to respiratory conditions (Kusiluka & Kambarage 1996).

Liver condemnation because of fasciolosis occurred in 4.5% of all cattle that were slaughtered. This prevalence is comparable to 6.7% found in Arusha (Mwabonimana *et al*. 2009). However, Mellau, Nonga and Karimuribo (2010a) found prevalence to be almost twice as high (8.6%) in the same area. A very high liver condemnation percentage as a result of fasciolosis (up to 30%) has been reported by Kamwela, Kassuku and Nonga (2013) in Sumbawanga, and Nzalawahe and Komba (2013) in Kigoma, Tanzania. Studies conducted in Ethiopia and Nigeria (Mohammed, Hailemariam & Mindaye 2012; Mulugeta, Begna & Tsegaye 2011; Njoku-Tony 2011) have also revealed higher occurrence of fasciolosis. This shows that fasciolosis is a large burden in cattle in most African countries. Although fasciolosis rarely cause mortalities in cattle, its effects result in reduced production and condemnation of livers during meat inspection in abattoirs (Kambarage *et al*. 1995).

Kidneys accounted for 13% of all organs and/or carcasses condemned. The main causes for kidney condemnation were congenital cysts and hydronephrosis. The results for hydronephrosis were similar to those reported by Mellau, Nonga and Karimuribo (2011) in Arusha, who found a prevalence of 1.9%. Renal calculi, nephritis, necrosis and melanosis were other causes of kidney condemnation although at very low rates. Both hydronephrosis and renal calculi were observed more often in the dry seasons. This was probably as a result of scarcity of water for animals, which predisposes animals to these renal conditions.

Pimply gut (oesophagostomosis) was the leading cause of condemnations of cattle intestines in the current study. Incidence was lower than reported by Mellau, Nonga and Karimuribo (2011) in Arusha and by Cadmus and Adesokan (2009) in Nigeria. In addition, the results indicated that pimply gut was observed more during the wet than the dry seasons, presumably as a result of favourable conditions for increased helminth activity in endemic areas. It is therefore imperative that control of helminths is stepped up during the wet season in order to reduce wastage of valuable meat from condemnations.

The results of this study have also shown the presence of hydatidosis in cattle, which is of public health importance and has an economic impact, especially in the rural poor communities where extensive grazing is practised (Berhe 2009; Ernest *et al*. 2009). These results are in agreement with Nonga and Karimuribo (2009), who found an infection rate of 4.2% in Arusha, Tanzania. However, higher prevalence was reported in Morocco, Ethiopia and Kenya (Azlaf & Dakkak 2006; Berhe 2009; Njoroge *et al*. 2002). Variations in prevalence of hydatidosis in cattle may be as a result of differences in the ecosystems, grazing patterns and status of echinococcosis in stray dogs, which are the definitive hosts. Because of its zoonotic importance, livestock extension and public health education need to be strengthened in order to reduce the disease burden.

The low prevalence of cysticercosis observed in the present study was in agreement with Mellau, Nonga and Karimuribo (2011), who found 0.3% in Arusha. However, these results were lower than those reported by Kebede (2008) in Ethiopia (18.5%). In addition, tuberculosis was found in 0.01% of slaughter cattle, which is within the range of 0.1% found by Mellau, Nonga and Karimuribo (2011) in Arusha and 0.3% in Tanga by Swai and Schoonman (2012). In spite of low prevalence recorded for the two zoonotic diseases, data suggest that they are endemic to Tanzania and therefore more concerted efforts to reduce their potential public health impact are needed.

Results from the present study revealed that the estimated financial loss resulting from organ and carcass condemnations was around $118 702 per annum. Liver condemnations alone accounted for a financial loss of $2706 per month, which is comparable with results obtained in Rukwa by Kamwela *et al*. (2013). Similarly, Mwabonimana *et al*. (2009) found financial loss as a result of fasciolosis to be $1169 in Arusha. The annual financial loss in the present study was comparable with results obtained from a study conducted in Nigeria (Cadmus & Adesokan 2009), which indicated an annual financial loss of $110 968. This loss of revenue by farmers, traders and the livestock industry has serious financial implications and a negative impact on the socio-economic wellbeing of those involved in the livestock value chain. Therefore disease control strategies should be implemented strictly.

The present study revealed that a high number of slaughtered cows were pregnant. The results were comparable to the findings by Maro (2014) in Tanga, Tanzania, who found that 40.5% of slaughtered cows were pregnant. Elsewhere, in the Western Province of Zambia, Zulu *et al*. (2013) reported 35.7% foetal wastage. However, our results indicated a much higher foetal wastage than the 8.6% reported in Arusha (Mellau, Nonga and Karimuribo 2011), as well as the 5% (Cadmus & Adesokan 2010) and 14.4% (Ardo *et al*. 2013) reported in Nigeria. The huge foetal wastage threatens growth of the livestock industry and therefore undermines government efforts to increase food production. Moreover, the slaughter of pregnant cows disregards animal welfare legislation (URT 2008). In addition, the quality of meat from pregnant cows may be reduced by the presence of increased hormonal levels in tissues.

## Conclusion

Generally, meat inspection in abattoirs provides useful information regarding the quality of meat and does not require many resources. On the other hand, abattoir surveys have limitations because diagnosis of diseases and conditions based on gross pathology has low sensitivity, so that some conditions are likely to be missed at routine meat inspection. The prevalence may also have been underestimated because of generally poor record keeping. Based on high organ condemnation rates observed during this study, effective extension, implementation and periodic review of routine livestock disease surveillance systems, including an effective trace-back system, are recommended. This will help to reduce the burden of diseases in animals. Furthermore, extension services to farmers should accompany pregnancy diagnosis in animals intended for slaughter at farm level in order to withdraw pregnant animals from slaughter. Moreover, strict enforcement of animal welfare legislation is advocated in order to curb the problem of slaughtering pregnant animals.
